# Comparison of effects of transcranial magnetic stimulation on primary motor cortex and supplementary motor area in motor skill learning (randomized, cross over study)

**DOI:** 10.3389/fnhum.2014.00937

**Published:** 2014-11-20

**Authors:** Yong Kyun Kim, Sung Hun Shin

**Affiliations:** ^1^Department of Physical Medicine and Rehabilitation, Myongji Hospital, Kwandong University College of MedicineKyunggi, South Korea; ^2^Department of Physical Medicine and Rehabilitation, Kyung Hee University College of MedicineSeoul, South Korea

**Keywords:** transcranial magnetic stimulation, supplementary motor area, learning, implicit memory, motor performance

## Abstract

Motor skills require quick visuomotor reaction time, fast movement time, and accurate performance. Primary motor cortex (M1) and supplementary motor area (SMA) are closely related in learning motor skills. Also, it is well known that high frequency repeated transcranial magnetic stimulation (rTMS) on these sites has a facilitating effect. The aim of this study was to compare the effects of high frequency rTMS activation of these two brain sites on learning of motor skills. Twenty three normal volunteers participated. Subjects were randomly stimulated on either brain area, SMA or M1. The motor task required the learning of sequential finger movements, explicitly or implicitly. It consisted of pressing the keyboard sequentially with their right hand on seeing 7 digits on the monitor explicitly, and then tapping the 7 digits by memorization, implicitly. Subjects were instructed to hit the keyboard as fast and accurately as possible. Using Musical Instrument Digital Interface (MIDI), the keyboard pressing task was measured before and after high frequency rTMS for motor performance, which was measured by response time (RT), movement time, and accuracy (AC). A week later, the same task was repeated by cross-over study design. At this time, rTMS was applied on the other brain area. Two-way ANOVA was used to assess the carry over time effect and stimulation sites (M1 and SMA), as factors. Results indicated that no carry-over effect was observed. The AC and RT were not different between the two stimulating sites (M1 and SMA). But movement time was significantly decreased after rTMS on both SMA and M1. The amount of shortened movement time after rTMS on SMA was significantly increased as compared to the movement time after rTMS on M1 (*p* < 0.05), especially for implicit learning of motor tasks. The coefficient of variation was lower in implicit trial than in explicit trial. In conclusion, this finding indicated an important role of SMA compared to M1, in implicit motor learning.

## Introduction

Motor skills like typing and hitting the keyboard require quick visuomotor coordination, and fast as well as accurate sequential finger movements. These motor skills can be learned either explicitly, practice after seeing the manuscript or implicitly, after memorizing manuscript and then self initiating its execution. Motor performance can be measured by visuomotor response time (RT), movement time, and accuracy (AC).

Repetitive transcranial magnetic stimulation (rTMS) is a safe, painless, and non-invasive method to modulate cortical excitability by stimulating the cerebral cortex (Rossini et al., 1994). A recent TMS study showed an effective connection between SMA and M1. The successful activation of this connection is likely mediated *via* excitatory interneurons (Arai et al., 2012).

It is well known that the primary motor cortex (M1) and supplementary motor area (SMA) are related in learning motor skills, especially movement sequencing (Tanji, 2001). Gerloff et al. observed that 20 Hz rTMS over the SMA induced AC errors in complex sequential movements (Gerloff et al., 1997). This finding indicated a critical role of SMA in the organization of forthcoming movements in complex motor sequences that are rehearsed from memory and fit into a precise timing plan. Similarly, there are several reports that both M1 and SMA have a key role in performing complex movements. In a positron emission tomography (PET) study, practice related increases were found in SMA and M1. This finding implied involvement in learning and storing the movement sequence, in a sequential maze learning task (van Mier et al., 2004).

In motor learning, M1 might contribute to optimizing the timing of visuomotor processing (Grafton et al., 2002). Applying anodal transcranial direct current stimulation (tDCS) to M1 appears to have a task-dependent effect on learning and memory formation (Saucedo Marquez et al., 2013). M1 is involved in the early consolidation of motor skills (Muellbacher et al., 2002). Instead, SMA might contribute to the preparation and execution of learned motor sequences (Shibasaki et al., 1993). SMA seems to play an important role in linking cognition to action (Nachev et al., 2008).

Several studies have demonstrated that M1 and SMA all appear to be particularly important in the early stage of motor skill acquisition previously (Platz et al., 2012; Sosnik et al., 2014). However, direct head to head comparison of these two brain sites for executing complex motor skill has not been addressed. Therefore, the goal of this study was to compare effects of high frequency rTMS on M1 and SMA, in learning motor skills. The motor skills comprised 2 modes of sequential finger movements: explicit motor learning and implicit motor learning; and were evaluated by the standards of motor performance which are RT, Movement time, and AC using the cross over design. Subsequently, we determined whether improved motor performance was related to the brain cortical activity or not, as measured by recruitment curve.

## Materials and methods

### Subjects

Twenty three naïve right handed healthy subjects participated in the study. The study group comprised 9 men, and 14 women. The average age was 24 ± 5.0 (mean ± standard deviation). All subjects gave their written informed consent to participate in the study. The study was approved by Institutional (Myongji Hospital) Review Board. Participation criteria included a normal neurological examination, no TMS contraindication, not being an active musician, and the ability to perform and learn the motor tasks.

### Motor task and experimental design

**Figure 1:**
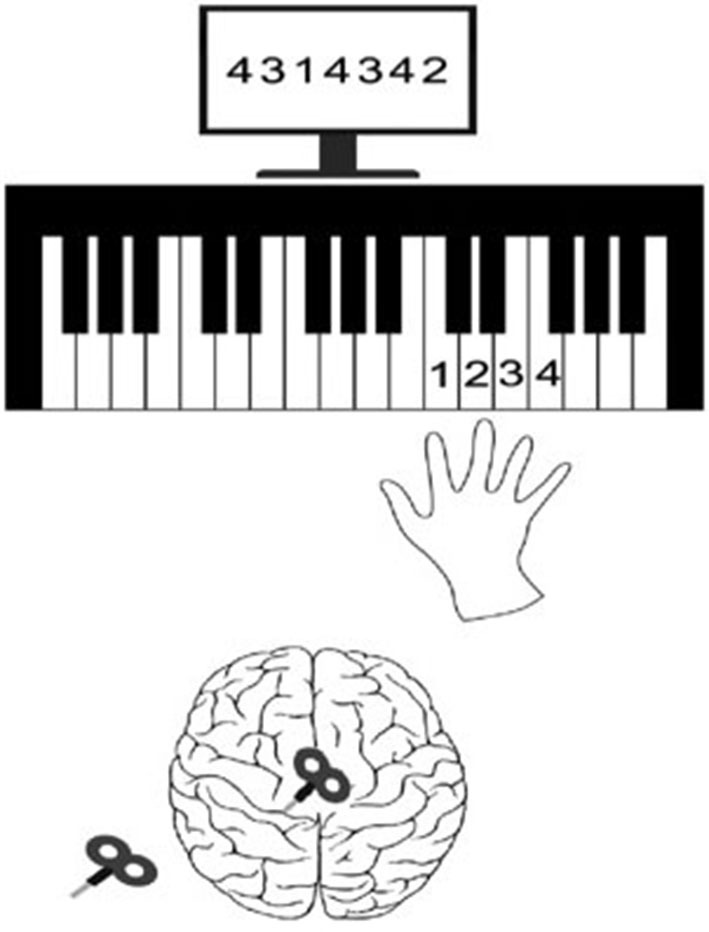
**Experimental setup**. A 7-digit sequence of numbers, which was a combination of 1, 2, 3, or 4 in a random order, was displayed on the monitor for 2500 ms. Participants were instructed to repeatedly push the 1, 2, 3, or 4 numbered buttons as accurately and quickly as possible with their right fingers. Each button was labeled with a number representing the finger: 1, 2, 3, and 4 represented the index, the middle, the ring, and the little finger, respectively. This task was repeated before and after high frequency rTMS on randomly assigned M1 or SMA. A week later, the same task was repeated again before and after rTMS on the other area at this time (cross- over study design).

**Figure 2:**
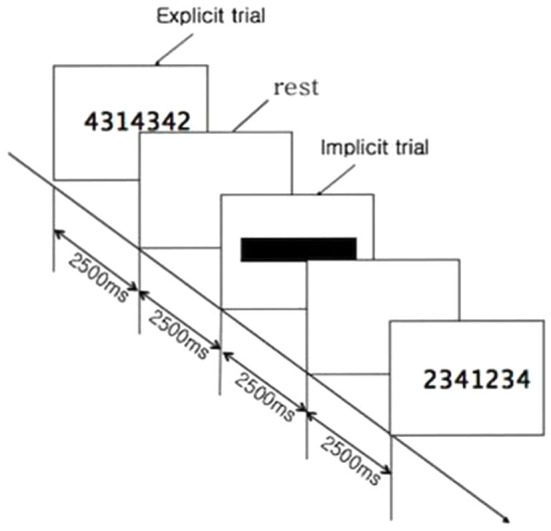
**Time sequence of motor task**. When combination of numbers appeared, participants were instructed to hit the 1st number as quickly as possible and push the remaining 6 numbered buttons for 2500 ms, which was explicit trial. It was followed by a rest for 2500 ms. After a rest, the numbers appeared again, but at this time they were blocked by a black bar. Subjects were instructed to strike the 7-digit sequence of numbers as quickly and accurately as possible, on the basis of memory for 2500 ms. That was defined as the implicit trial. The 2500 ms × 4 cycle was repeated 8 times. A 20 s rest followed. This set was repeated 8 times.

Normal volunteers performed a block of sequential finger-tapping tasks. The sequential visuomotor task paradigm involved repetitive push-button action in response to a 7-digit number stimulus displayed on a computer screen. The subjects were seated 70 cm in front of a 21 inch monitor. The 7 digit sequence of numbers consisted of a combination of 1, 2, 3, or 4 in random order, and was displayed at the center of the monitor for 2500 ms (ms). Participants were instructed to repeatedly push the 1, 2, 3, or 4 numbered buttons as accurately and quickly as possible, with their right fingers. Each button was labeled with a number representing the finger to be used: 1, 2, 3, and 4 represented the index, the middle, the ring, and the little finger, respectively (Figure 1). When the combination of numbers appeared, participants were instructed to hit the 1st number as quickly as possible and push the remaining 6 numbered buttons for 2500 ms. That was defined as the explicit trial. After a 2500 ms rest, the numbers appeared again, but at this time they were blocked by a black bar. The subjects were instructed to strike the 7 digit sequence of numbers as quickly and accurately as possible on the basis of memory for 2500 ms. That was defined as the implicit trial. It was followed by a rest for 2.5 s (Figure 2). This 2500 ms × 4 cycle was repeated 8 times. A 20 s rest followed. This entire set was repeated 8 times. Total duration of the motor task was 13 min. There were sessions of practice prior the actual test, when subjects rehearsed this visuomotor task for 5 min.

### Evaluation of motor performance: response time (RT), task duration (TD), and accuracy (AC)

**Figure 3:**
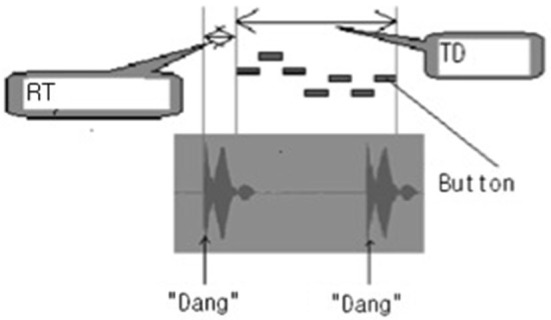
**Measurements of task duration (TD) and response time (RT) on the Musical Instrument Digital Interface (MIDI) program**. When the combination of numbers appeared, a “Dang” sound was heard; and when the combination of numbers disappeared, a “Dang” sound was heard. Between the two “Dang” sounds, 7 pressed buttons were expressed as 7 bars. TD represented the time interval between the start of pressing the first button and the end of pressing the last button. RT was the time interval from the onset of display of combination of numbers to pressing the first button. It was expressed in millisecond (ms).

The motor performance was determined by assessing TD, RT, and AC using a keyboard Musical Instrument Digital Interface (MIDI) program. TD represented time interval required to complete the motor task. It was defined as the time interval between the start of pressing the first button and the end of pressing the last button. It was expressed in millisecond (ms). RT was the time interval between observing the 7-digit sequence of numbers and pressing the first button (Figure 3). AC was the total number of correctly pressed buttons.

### Evaluation of motor cortex excitability: motor threshold (MT) and recruitment curve (RC)

MT and RC represented motor cortex excitability. MT and RC were measured before the motor task and after high frequency rTMS, respectively. The resting MT (RMT) was defined as the lowest stimulation intensity required to evoke a motor evoked potential (MEP) in the relaxed first dorsal interossei (FDI) of > 50 μV in 5 out of 10 trials. RC was obtained at 5 stimulus intensities i.e., 100%, 110%, 120%, 130% and 140% of RMT. Ten trials of TMS per intensity were performed. Peak-to-peak MEP amplitudes were measured and plotted against stimulus intensity, thus giving a RC on the contralateral hemisphere in each subject. MT and RC were assessed before and after 20 Hz rTMS on the randomly assigned M1 or SMA of the left cerebral hemisphere, respectively. The trial was repeated after a week, in which 20 Hz rTMS was delivered on the other area respectively (by the cross over study design).

### Intervention: high frequency (20 Hz) stimulation on primary motor cortex (M1) and supplementary motor cortex (SMA)

Participants were seated in a reclining chair with their arms and hands relaxed. The 20 Hz rTMS was performed through a MagPro^®^ X100 (Medtronics, USA) with a 70 mm figure of eight coil over the two target areas of the brain cortex, i.e., M1 or SMA randomly.

#### High frequency rTMS on M1

The coil was placed tangential to the scalp, with the handle pointing 45° posterolaterally to stimulate the motor cortex. A white swimming cotton cap with a pre-marked grid (spacing 1 cm in latitude and longitude) was tightly fitted to the subject’s head size and the center was matched with the scalp vertex (Cz) as defined by 10–20 International System for EEG electrodes. TMS was performed by moving the coil by 1 cm steps around the presumed hand motor area to determine the optimal position for the activation. Once the optimal position was found, the “hot spot” was marked on a cap. MEPs were recorded from paired 10 mm stainless steel disk electrodes, with the active electrode placed on the right FDI muscle belly and the reference electrode on the 2nd metacarpophalangeal joint. rTMS was delivered over M1 at a frequency of 20 Hz at 0.9 of RMT. The total number of TMS pulses was 1200.

#### High frequency rTMS on SMA

Previously described criteria were used to determine the site for SMA stimulation (Matsunaga et al., 2005) i.e., the optimal position for activation of the right tibialis anterior (TA) muscle, by moving the coil in 1 cm incremental steps along the midline around the scalp vertex (Cz) with the handle pointing 90° to the left. The active MT (AMT) was determined as the lowest stimulation intensity required to evoke a MEP > 200 μV in 5 out of 10 trials at 20% of maximal voluntary contraction (MVC) of TA. Stimuli of ~1.3 AMT were given by moving the coil anteriorly along the midline in 1 cm steps. The SMA was defined as being 1 cm anterior to the last site from which MEPs could be evoked during the contraction (Perez et al., 2008). rTMS was delivered over the SMA at a frequency of 20 Hz at 0.9 of AMT. The total number of TMS pulses was 1200.

### Data analysis

Paired *T*-test was used to assess changes of motor performance before and after high frequency stimulation on M1 or SMA. To compare effects of rTMS according to application brain site (M1 vs. SMA) on motor learning (explicit or implicit), two-way ANOVA (stimulation site × time) was used. And to compare effects of rTMS on performance variability, we used the coefficient of variation (CV, CV = SD/mean) for the RT, movement time on motor performance in both explicit and implicit trials. This would provide the information about the effect of rTMS on the dispersion of the motor performance data with respect to the corresponding mean. Significance was set at *p* < 0.05. Statistical analysis was conducted with PASW Statistics 18.0 for Windows (SPSS, Inc. Chicago, IL, USA). All descriptive statistics are reported as mean ± SD.

## Results

### Coordinates of SMA

**Figure 4:**
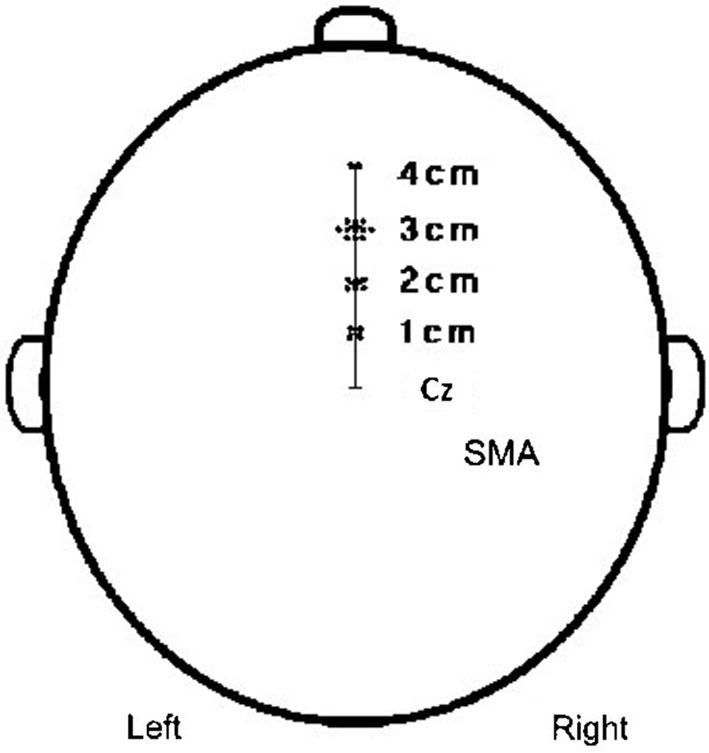
**Coordinates of SMA**. The coordinates of SMA were 2 cases; 4 cm to the anterior, 11 cases; 3 cm to the anterior, 6 cases; 2 cm to the anterior, 4 cases; and 1 cm to the anterior from the scalp vertex.

Twenty-three normal volunteers participated. Their sites for the SMA stimulation were determined to be 0~3 cm anterior from the optimal position for activation of the TA muscle, 1–4 cm anterior to Cz (Figure 4) in the 10–20 EEG system. Some subjects who had their own brain magnetic resonance image (MRI) were confirmed by guiding the TMS coil to a location defined by anatomical landmarks on the individual’s MRI.

### Motor performance

**Figure 5:**
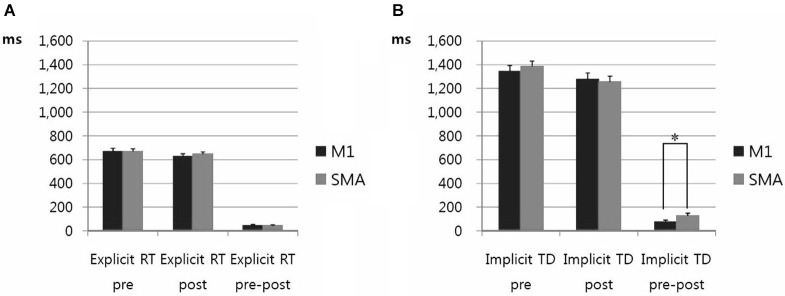
**(A)** Response time of motor performance in explicit trials. RT of visuomotor task in explicit trials was significantly shortened after high frequency rTMS vs. pre-stimulation in case of stimulation on the M1 and the SMA. There was not a significant decrease in RT between high frequency rTMS on M1 and SMA. Error bars depict standard errors of the mean.** (B)** Task duration of motor performance in implicit trials. TD of memorized motor task was significantly shorter after high frequency rTMS vs. pre-stimulation in case of stimulation on the M1 and the SMA. The amount of shortened TD post-stimulation on SMA was significantly increased compared to TD post-stimulation on M1. * *p* < 0.05. Error bars depict standard errors of the mean.

No carry over effect was observed for all 3 measures i.e., AC, RT, and TD. AC was steadily maintained before and after stimulation (data not shown). RT of explicit learning of visuomotor task was significantly shorter post-stimulation vs. pre-stimulation. But the magnitude of decrease in RT was not different between rTMS on M1 vs. SMA (*F*_(9,29)_ =3.583, *p* = 0.062) (Figure 5A). TD of implicit learning of memorized motor task was significantly shorter post-stimulation vs. pre-stimulation. There was a significant decrease in TD after stimulation on SMA vs. TD after stimulation on M1 (*F*_(3,5)_ = 10.016, *p* = 0.02) (Figure 5B).

**Figure 6:**
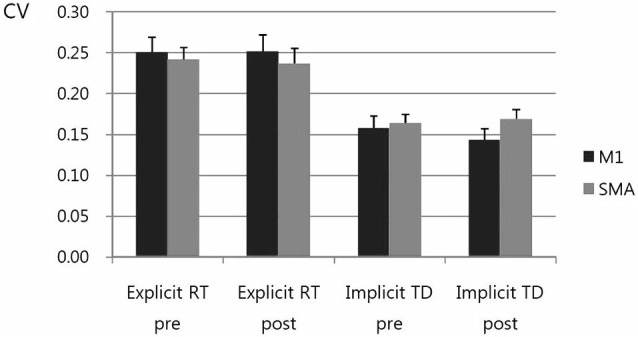
**Coefficient of Variation (CV)**. CV = SD/mean for the response time (RT) on motor performance in explicit trials and for the movement time (TD) in implicit trials. The CV was higher in explicit trial than in implicit trial. The CV of RT in explicit trial of visuomotor task showed somewhat decreased, post-stimulation vs. pre-stimulation, but not significant. Also, the magnitude of decrease in CV of RT was not different between rTMS on M1 vs. SMA. The CV of TD in implicit trials was not different between post-stimulation vs. pre-stimulation. Also, the magnitude of changes was not different between rTMS on M1 vs. SMA.

We investigate the effects of rTMS on performance variability by calculating and comparing into the ANOVA the coefficient of Variation (CV). CV = SD/mean for the RT on motor performance in explicit trials and for the movement time (TD) in implicit trials. The CV of RT in explicit trial of visuomotor task showed somewhat decreased post-stimulation vs. pre-stimulation but not significant. Also, the magnitude of decrease in CV of RT was not different between rTMS on M1 vs. SMA. The CV of TD in implicit trials was not different between post-stimulation vs. pre-stimulation. Also, the magnitude of changes was not different between rTMS on M1 vs. SMA. (Figure 6).

### Cerebral cortex excitability

**Figure 7:**
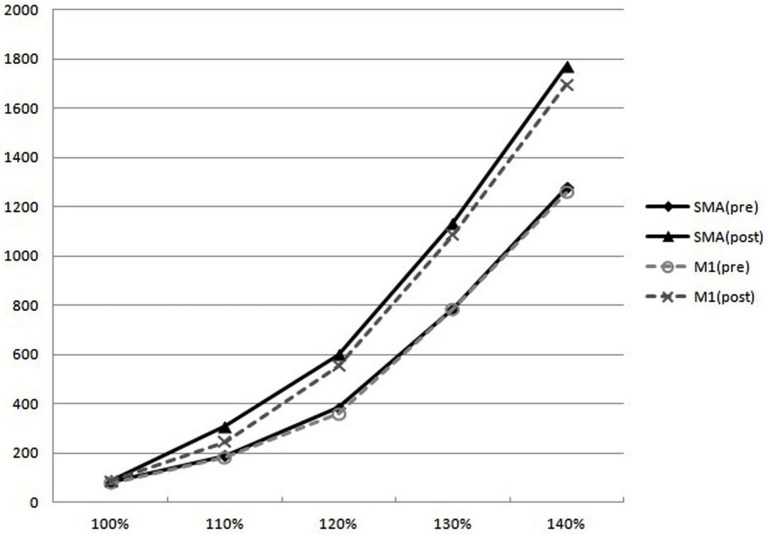
**Recruitment curves**. On RC, MEPs at the 110%, 120%, 130%, and 140% of the RMT increased post-stimulation on both M1 and SMA vs. pre-stimulation. But the amount of increased MEPs was not different in M1 vs. SMA.

MT was not different between stimulation on M1 and SMA (data not shown). On RC, MEPs increased post-stimulation on both M1 and SMA vs. pre-stimulation. The amount of increased MEPs was not different in M1 vs. SMA stimulation (Figure 7).

## Discussion

Twenty Hz rTMS on SMA resulted in a significant decrease in TD in implicit motor learning of a complex motor task (7-digit sequential finger movements) as compared with 20 Hz rTMS on M1. However, the RC showed no significant difference after rTMS between M1 and SMA in cortical excitability of primary motor cortex; even though increase in cortical excitability was observed after 20 Hz rTMS on both M1 and SMA.

The improvement of motor performance especially movement time was observed after 20 Hz rTMS on SMA compared to M1, in the implicit but not in the explicit trial. This finding supported the proposed theoretical role of the SMA. The SMA is thought to play a significant role in self-initiated movements rather than external cue-guided movements. This result was compatible with previous studies (Chouinard and Paus, 2010; Makoshi et al., 2011; Lu et al., 2012).

RT of visuo-motor performance was more largely variable, compared with movement time. CV was higher in explicit trial than in implicit trial. Previous study reported the explicit judgments had considerably larger variability than the implicit judgments (Rand and Heuer, 2013). That is line with this study. In contrast, recent work showed that motor task-concurrent dual tDCS on M1 resulted in significantly reduced RT by 23% in explicit motor learning (Karok and Witney, 2013). In this study, offline stimulation, that is stimulation applied during the interval between motor tasks, was used. On line stimulation and tDCS might cause different effects.

Motor learning can be expressed by two ways. One is the more decreased mean value and the other is the narrower dispersion of data with respect to the corresponding mean. In this study, CV was not different in post-stimulation vs. pre-stimulation, and between rTMS on M1 vs. SMA. However, the mean value of movement time significantly decreased after 20 Hz rTMS on SMA compared to M1, in the implicit trial. That might be explained the dispersion already narrowed, thus performance gains expressed by the magnitude of mean value. That might be related to the characteristics of high frequency (20 Hz) rTMS, which would be associated with the higher neuronal efficiency (Sosnik et al., 2014).

For the neuronal efficiency of the brain, another fMRI study showed that on Serial Interception Sequence Learning (SISL) task, decreased activity across a cortical network may reflect improved efficiency in motor planning and execution for the trained sequence, especially non-declarative skill learning (Gobel et al., 2011). Our findings were supported by these previous reports.

One of the possible explanatory mechanisms for this finding would be that the numerous neural connections between SMA and M1, especially the excitatory interneuron, may be activated after 20 Hz stimulation (Raux et al., 2010; Arai et al., 2012). On the recruitment curve, the excitability of the primary motor cortex increased post-stimulation on the SMA. Recent studies reported that short-interval intracortical facilitation (SICF), which reflected the facilitatory circuit within the M1, increased after delivering rTMS on the SMA (Arai et al., 2012; Shirota et al., 2012). We accordingly expected that motor cortex excitability would be larger after 20 Hz rTMS on the SMA, rather than on the M1. However, the changes in the excitability of the corticospinal tract were not significantly different between stimulation on the M1 and the SMA. Therefore, this result was in agreement with the proposition from a previous study that skilled learning lead to the development of specialized neural circuits, which allowed the execution of fast sequential movements without average increases in brain activity on functional MRI (Gobel et al., 2011; Wiestler and Diedrichsen, 2013).

Another possible explanatory mechanism could be the activation of cortico-subcortical neural circuitry (Bestmann et al., 2005; Gheysen et al., 2011; Censor et al., 2014). SMA receives afferent input from basal ganglia. In previous report, sequence-specific motor learning was reliant upon striatum activation, especially for the motor task requiring the increased speed of movement (Wadden et al., 2013).

Reviewing the previous articles on TMS stimulation of SMA for modulating motor performances, it is evident that the timing of stimulation affects results, especially the performance of complex motor skills. Late TMS interference inhibited motor performance, whereas early TMS interference facilitated motor performance (Gregori et al., 2005). Motor performance was affected by not only the timing of interference, but also by the intensity and the stimulation site of TMS. Particularly for movement time, intensity is less crucial than the stimulation site (Gregori et al., 2005). Our finding that early TMS interference on SMA facilitated motor performance, especially in the movement time is in keeping with this previous report.

AC of motor performance was maintained steadily regardless of stimulation site and time interval. So, it can be assumed that the attention level was constantly maintained during the study. A previous study reported that in the complex sequence learning, the stimulation of both M1 and SMA induced AC errors. Therefore these two areas would play a critical role in the organization of forthcoming movements in complex motor sequences, that are rehearsed from memory and fit into a precise timing plan (Gerloff et al., 1997; Wymbs and Grafton, 2013).

SMA occupies a higher level in the motor hierarchy than M1. Activity in SMA precedes any changes in M1. SMA is one of a several higher order motor cortical areas, that lie along the medial side of the frontal cortex (Makoshi et al., 2011). A recent study revealed an increase of automatic imitation of observed movements following 5 Hz rTMS on SMA (Finis et al., 2013). This finding supported the concept that the SMA could contribute to the preparation and execution of learned motor sequences by contributing to encoding and planning the next element in a motor sequence (Perez et al., 2008). This is compatible with our finding in implicit trial.

In this study 20 Hz high frequency rTMS on both the M1 and the SMA improved motor performance, by shortened RT in the explicit trial and movement time in the implicit trial compared to pre-stimulation. Our previous work showed when sham stimulation was applied either on M1 or SMA, there were no significant differences between post-stimulation and pre-stimulation motor performance measures, visuo-motor RT, TD, and AC (*N* = 16, data not shown). Therefore, we considered that 20 Hz high frequency rTMS on M1 and SMA could improve motor learning *via* increase of cortical activity. This effect did not last for more than a week. Further study is required for the long-term effects of repeated stimulation, in the future.

A previous study with MRI-navigated TMS of SMA, reported that the exact site of SMA was within 3 cm to the front, from the vertical line from the anterior commissure, perpendicular to the anterior-posterior commissure line in the sagittal plane (Mayka et al., 2006). Additionally, it was reported that SMA was located within 1–4 cm, mostly 2–3 cm from Cz on the EEG 10–20 system (Hikosaka et al., 1996; Lee et al., 1999). In our study, the SMA in 17 out of 23 subjects was situated within 2–3 cm from Cz. The SMA location was confirmed in some subjects (who had their own MRI) using a MRI-guided navigation system.

In conclusion, this study showed that 20 Hz high frequency rTMS on SMA rather than M1, significantly reduced movement time in the implicit learning task of sequential finger movement. Further study is required to understand the details of the underlying mechanism(s), using functional imaging. This finding is applicable to learning motor skills like performing musical instruments and typing which require accurate and fast finger movements. The findings may also be applicable in brain injured patients, since the SMA may be one of the important brain areas for executing automatic independent movements in self care activities.

## Conflict of interest statement

The authors declare that the research was conducted in the absence of any commercial or financial relationships that could be construed as a potential conflict of interest.
